# Non-Mutually Exclusive Deep Neural Network Classifier for Combined Modes of Bearing Fault Diagnosis

**DOI:** 10.3390/s18041129

**Published:** 2018-04-07

**Authors:** Bach Phi Duong, Jong-Myon Kim

**Affiliations:** School of Electrical, Electronics and Computer Engineering, University of Ulsan, 44610 Ulsan, Korea; duongbachphi@gmail.com

**Keywords:** bearing fault diagnosis, combined mode classification, deep neural network, stacked denoising autoencoder

## Abstract

The simultaneous occurrence of various types of defects in bearings makes their diagnosis more challenging owing to the resultant complexity of the constituent parts of the acoustic emission (AE) signals. To address this issue, a new approach is proposed in this paper for the detection of multiple combined faults in bearings. The proposed methodology uses a deep neural network (DNN) architecture to effectively diagnose the combined defects. The DNN structure is based on the stacked denoising autoencoder non-mutually exclusive classifier (NMEC) method for combined modes. The NMEC-DNN is trained using data for a single fault and it classifies both single faults and multiple combined faults. The results of experiments conducted on AE data collected through an experimental test-bed demonstrate that the DNN achieves good classification performance with a maximum accuracy of 95%. The proposed method is compared with a multi-class classifier based on support vector machines (SVMs). The NMEC-DNN yields better diagnostic performance in comparison to the multi-class classifier based on SVM. The NMEC-DNN reduces the number of necessary data collections and improves the bearing fault diagnosis performance.

## 1. Introduction

Reducing the cost of maintenance and decreasing the shutdown time are important components of maintenance in modern industries. Faults in the rolling element bearings are the most common cause of rotary-machine breakdown. According to research by the Electric Power Research Institute [1], bearing failures account for 40% to 50% of faults in induction motors alone. These unscheduled breakdowns can cause an entire system to crash, thereby leading to tremendous economic losses. Thus, bearing fault diagnosis has received extensive attention for the prevention of unexpected downtime in industries and for ensuring safety while maintaining efficiency of the manufacturing environment. In addition, automatic fault detection of bearings using sensors and remote control stations can also be helpful in the reduction of labor costs [2]. 

Bearing failures can be categorized into two types: single mode faults and combined mode faults. In single mode faults, only one bearing component develops a defect, i.e., the inner raceway (BCI), outer raceway (BCO), or roller element (BCR). Defects that simultaneously occur in more than one component are known as combined mode faults. They include the inner–roller defect (BCIR), outer–inner defect (BCOI), outer–roller defect (BCOR), and outer–inner roller defect (BCOIR). When a bearing has combined mode defects, i.e., defects in multiple components, then the fault identification and classification becomes more difficult, owing to the complexity of the recorded signals that are used for the diagnosis of these faults. Consequently, an efficient method is required for the detection of combined multiple defects in bearings.

The fault detection and diagnosis methods include data-driven, model-based, and hybrid techniques. Hybrid techniques are usually a combination of model-based and data-driven methods. In [3,4] model-based methods along with fuzzy filters have been proposed to ensure robustness and sensitivity to fault condition. Similarly, in [5,6] model-based methods have been effectively used for the detection of faults in different systems. With the help of multi-sensor systems, data-driven techniques are becoming more popular and robust in the detection of faults in industrial environments [7,8]. In recent years, machine learning has led to significant advancements in bearing fault diagnosis of rotary machines. These techniques offer better adaptability to complex systems and do not require any physical model for bearings. However, sufficient historical data is required for constructing a model for the measurement data, which is acquired through a variety of sensors and measuring equipment. Many machine learning techniques have been applied to diagnostics in bearings using vibration and acoustic emission data. These techniques include Bayesian inference [9], principal component analysis [10], decision trees [10], hidden Markov models [11], artificial neural networks (ANN) [12,13], k-nearest neighbor based methods (K-NN) [14], and support vector machines (SVM) [15]. Kang et al. [14] and Nguyen et al. [15] proposed effective fault diagnostic schemes, which involve feature pool configuration techniques to select and extract the best features from both single and combined modes. The extracted features, which contain rich information on each type of bearing fault, are provided to machine learning algorithms for classification. To use these features for multi-fault diagnosis, Islam et al. [16] proposed a Bayesian inference-based multi-class SVM, which is effective in diagnosing multiple fault conditions of the bearing. 

In the above algorithms, data for each fault mode is required for developing models that can then be used to distinguish different faults. Thus, without data for all types of faults, i.e., both single and combined faults, these techniques are not able to effectively distinguish all faults. However, we hypothesize that given data on the single fault modes of a bearing and an effective machine learning technique, we should be able to detect multiple combined faults without requiring separate measurement data for those multiple combined faults. Hence, to achieve this objective, a method based on a deep neural network (DNN) non-mutually exclusive classifier (NMEC) aggregating stacked denoising autoencoders (SDAE) is proposed in this paper. NMEC-DNN can automatically extract useful features and thereby gather useful insights from the measurement data on each fault type. These extracted features are high-level representations of the original signals and can appear as abbreviated fault information by removing the residual part.

The main contribution of this research is the NMEC-DNN strategy of recognizing and classifying the combined bearing defects. In the proposed method, measurement data for only the normal state and single-fault modes of bearings is used to train the DNN, which is then used to diagnose both single and combined faults in bearings. The DNN is pre-trained using a stacked denoising autoencoder to extract rich information features from each sample. Then, the NMEC is used to classify the unknown samples. This methodology results in significantly better diagnostic performance while reducing the number of scenarios for data collection. The proposed NMEC-DNN is also compared with the conventional multiclass SVM.

The remainder of this paper is structured as follows. Section 2 outlines the background of different kinds of fault mode signatures and the support vector machine method. The NMEC-DNN architecture is presented in Section 3. The results and discussion are given in Section 4. Finally, the conclusions are provided in Section 5.

## 2. Basic Concepts of Bearing Fault Diagnosis

The core concept of this research is the intrinsic relationship among different bearing faults and the signatures that can be used to diagnose these faults. This section describes this relationship. It additionally presents SVM, which is the method implemented for comparison with the proposed method.

### 2.1. Bearing Fault Signature

**Figure 1:**
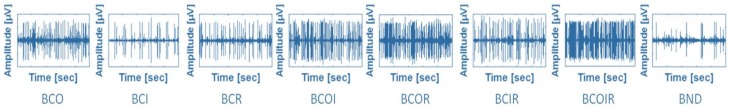
Acoustic emission signal for each bearing condition.

A bearing fault, usually referred to as a localized defect is caused by many factors, such as material fatigue, corrosion, and poor lubrication. Material fatigue, due to the cyclic loading of bearings, can lead to the development of spalls, which can grow into cracks. When the cracks appear on the surface of bearing components, they can lead to accelerated bearing degradation due to pitting and tearing of the bearing material. Moreover, inadequate lubrication increases friction, which leads to increased metal-to-metal contact and greater plastic deformation due to local contact phenomenon. This increases the temperature at the local point, further accelerating the bearing deterioration process. Furthermore, high humidity in the environment also acts as a catalyst for bearing corrosion [17]. When the defect emerges in the bearing, it induces an anomalous pattern in the vibration amplitude. Contacts between elements in the damage zones generate high-level, short-duration impulse forces. For example, a defect on the outer or inner raceway produces impulses when the roller (or ball) passes over the defect zone. Since the machine rotation is periodic, these impulses tend to be generated periodically. The frequency of these impulses is known as the defect frequency (or bearing characteristic frequency) and is determined by the location of the defect and the geometry of the bearing. These impulses stimulate high frequency resonances in the bearing, which act as carrier signals and are amplitude-modulated by the defect frequencies [18]. The existence of a particular localized defect is indicated by the occurrence of impulses at a specific defect frequency. Moreover, the amplitudes of these impulses can provide information on the degree of fault severity. Figure 1 depicts the acoustic emission signal for different types of bearing defects.

Compared to the signal of the no-defect bearing (BND), signals for faulty bearings show abnormal impulses. The signals for bearings with combined bearing defects, such as BCOI, BCOR, BCIR, and BCOIR, show more complicated impulse patterns as compared to the single bearing defects, such as BCO, BCI, and BCR. The single bearing defects such as outer race fault, inner race fault and roller fault can be detected using the ball-pass frequency of the bearing outer-race (BPFO), the ball-pass frequency of the inner-race (BPFI), and twice the ball-spin frequency (2×BSF; i.e., the defect point on the roller hits the outer and inner raceways to produce two impulses in each roller’s spin cycle), respectively [19]. These frequencies can be calculated using (1)–(3).

(1) $$BPFO=\frac{nf_{r}}{2}\left(1-\frac{d}{D}\cos\phi \right)$$

(2) $$BPFI=\frac{nf_{r}}{2}\left(1+\frac{d}{D}\cos\phi \right)$$

(3) $$BSF=\frac{Df_{r}}{2d}\left[1-{\left(\frac{d}{D}\cos\phi \right)}^{2}\right]$$

Here, $f_{r}$ is the shaft rotation speed, n is the number of rolling elements, ϕ denotes the contact angle (or angle of the load from the radial plane), d represents the roller diameter, and D is the pitch diameter. These cyclic impulses appear in the low-frequency range, and they are amplitude-modulated to higher frequencies. Because of the modulation of the carrier signal, envelope analysis [20] is applied as a demodulation technique to detect these impulses by isolating the impulse signal from the carrier signal. 

As mentioned above, the defect frequencies modulate the amplitude of the carrier frequencies. The amplitude modulation occurs when a defect on the moving parts of bearing, i.e., the inner race and rollers, periodically enters and leaves the load zone. Because of this amplitude-modulation, the defect frequencies cannot be detected in the spectrum of the raw AE signals. This is due to two reasons; firstly, the spectrum of the raw AE signals is flooded with the high magnitude resonance frequencies and measurement noise, making it very difficult to detect the low magnitude defect frequencies. Secondly, the BPFI and 2×BSF do not appear in the spectrum of the raw AE signal, rather they appear as sidebands of the carrier frequency, making the detection of the inner and roller faults very difficult using the spectrum of the raw AE signal. It is pertinent to mention here that the defect frequencies are in tens of Hertz, whereas the resonance frequencies range from several kilohertz to tens of kilohertz. The amplitude modulation results in sidebands in the spectrum of the raw AE signal that are spaced apart by the modulation frequency, i.e., the BPFI or 2×BSF, and centered about the carrier frequency. It is very difficult to recover BPFI and 2×BSF from these sidebands and thereby use them to detect inner race and roller faults. Thus, the simple fast Fourier Transform (FFT) of the raw time domain signal is not very useful for fault detection in bearings. Rather, first the raw AE signal needs to be demodulated to recover the envelope signal, which can then be analyzed to detect the defect frequencies. Hence, to remove the carrier signal, first, the Hilbert transform [21] is used to convert the time-domain signal to the time-frequency domain as $\tilde{x}(t)=H[x(t)]$, where $\tilde{x}(t)$ is formed from x(t) in the Hilbert space. Later, the complex analytic signal is calculated by $c(t)=x(t)+i\tilde{x}(t)$, where $i^{2}=-1$ is the imaginary unit. The equation of $e(t)=\left|c(t)\right|=\sqrt{x^{2}(t)+{\tilde{x}}^{2}(t)}$ yields the envelope signal. The envelope signal is then analyzed using the FFT. Finally, the envelope power spectrum is obtained as the squared absolute value of the FFT signal. 

**Figure 2:**
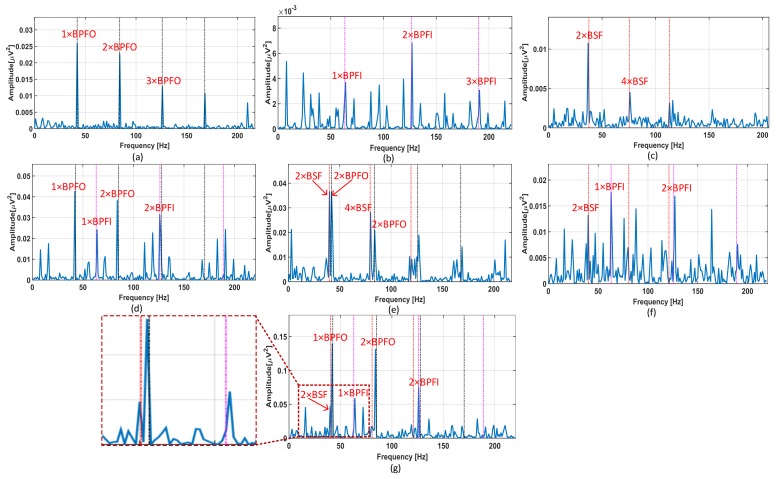
Envelop spectrum of seven types of fault signals: (**a**) Bearing-Crack-Outer; (**b**) Bearing-Crack-Inner; (**c**) Bearing-Crack-Roller; (**d**) Bearing-Crack-Outer-Inner; (**e**) Bearing-Crack-Outer-Roller; (**f**) Bearing-Crack-Inner-Roller; (**g**) Bearing-Crack-Outer-Inner-Roller.

Figure 2 shows the envelope power spectra of different bearing fault signals. From experience, we know that the bearing fault signatures of multiple concurrent defects differ in terms of the summation of the corresponding single defects. However, if the interaction between defect signals is adequately small, the bearing fault classes can be assumed as independent non-exclusive classes. In this study, the envelope spectrum of the combined defect (BCOI) (Figure 2d) contains both harmonics of BPFO and BPFI. Hence, the combined defect of BCOI can be treated as the combination of BCO (Figure 2a) and BCI (Figure 2b).

The same results are observed when analyzing the envelope spectra of other combined defects, such as BCOR (Figure 2e), BCIR (Figure 2f), and BCOIR (Figure 2g). A signal is classified as the combined fault class if its extracted features exhibit the characteristics of both the single faults that constitute the combined fault. Based on this property, the proposed NMEC-DNN is built to recognize the single fault components in the complex multi-fault signals.

**Figure 3:**
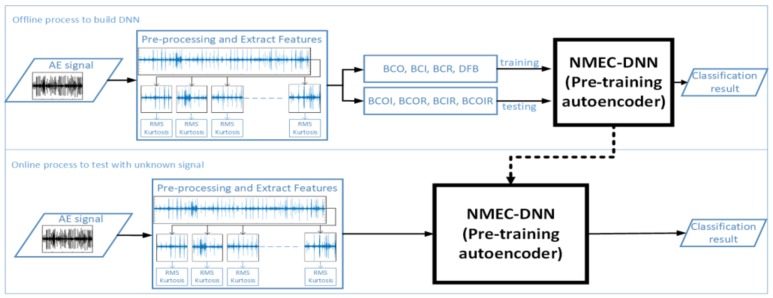
The process of classification by non-mutually exclusive classifier-deep neural network (NMEC-DNN).

**Figure 4:**
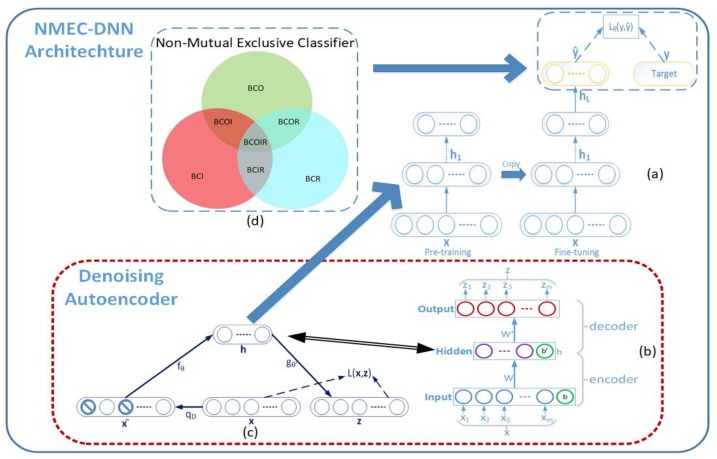
NMEC-DNN architecture: (**a**) DNN training process (**b**) Autoencoder structure (**c**) DAE structure (**d**) NMEC structure.

Figure 3 shows the processes involved in the proposed scheme for combined fault diagnosis. In the offline process, after the pre-processing step, data is separated into two parts: the first part contains the single fault samples, which are only used for training the NMEC-DNN; the second part contains the combined fault samples that are used for testing the network. In the online process, an unknown signal is placed in the network to be labeled as belonging to one of the eight fault classes. The architecture of NMEC-DNN is shown in Figure 4 and presented in Section 3.

### 2.2. Multi-Class Support Vector Machine

The multi-class SVM is employed for comparison with the proposed method. Among the SVM architectures, the kernel SVM [22] is a kernel based method for building classification models. This method is fundamentally a binary classification technique that separates two classes using a maximum-margin hyperplane in a hyper dimensional feature space as discussed in detail in [23]. 

**Figure 5:**
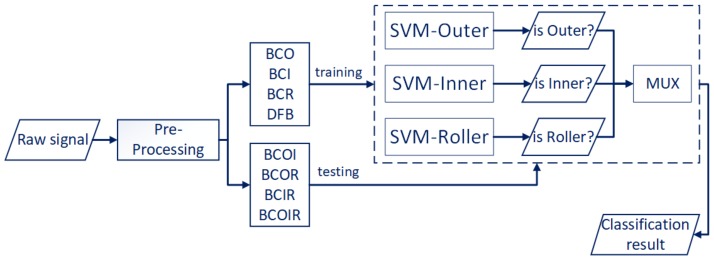
Set of support vector machines (SVMs) for classification.

In this paper, the multi-class SVM with the one-against-all (OAA) strategy is used for bearing fault diagnosis in the same setting as the proposed method NMEC-DNN for an even comparison of the two techniques. The multi-class SVM is trained using the normal and single defect signals and then tested using the multiple combined defect signals. This is illustrated in Figure 5, which shows that the SVM is trained to classify three fault types (outer, inner, roller) and the normal state. The OAA-SVM framework transforms this four-class classification problem into three two-class classification sub-problems. They are denoted by “SVM-outer,” which determines whether a signal belongs to an outer or non-outer fault (normal, inner, roller). “SVM-inner” determines whether a signal belongs to an inner or non-inner fault (normal, outer, roller). In addition, “SVM-roller” determines whether a signal belongs to a roller or non-roller fault (normal, inner, roller). The outputs of the three SVMs are binary values (zero or one), and a multiplexer is used to gather and interpret the outputs to infer the health of the bearing.

## 3. The Proposed Combined Bearing Fault Diagnosis Methodology

Figure 4 illustrates the processes involved in the proposed method for NMEC-DNN training and testing. The network training is done in two phases, i.e., a pre-training phase, and fine-tuning phase, as shown in Figure 4a.

### 3.1. Pre-Training Phase with a Stacked Denoising Autoencoder

The autoencoder is a type of ANN that finds a nonlinear mapping to transfer input x in dimension d to a new representation, h, in dimension $d^{\prime }$ so that $h=f_{\theta }(x)=s(Wx+b)$, where θ={W,b}, W is a $d^{\prime }\times d$ matrix of synapse weights, b is a bias vector of dimension $d^{\prime }$, and s(⋅) is a nonlinear mapping. The autoencoder learning procedure consists of two steps: encoder and decoder. The encoder finds a deterministic mapping, $f_{\theta }$, as above. The decoder maps back the representation h to reconstruct the vector $z=g_{\theta^{\prime }}(h)=\varphi (W^{\prime }h+b^{\prime })$, which has the same dimension as the vector x, where $\theta^{\prime }=\{W^{\prime },b^{\prime }\}$ and φ(⋅) is an affine or nonlinear mapping. 

Figure 4b illustrates the architecture of the original autoencoder. The encoder and decoder are simultaneously trained, and thus the object of reconstructed vector z is as close as possible to the input vector x. For this purpose, the network learns to minimize the distance, $L(x,z)={\left\|x-z\right\|}^{2}$ between the input vector and its learned representation. The result from the autoencoder training is vector h, which is the higher-level representation of input x. Depending upon the dimension $d^{\prime }$, a different representation of the vector is formed. However, when the dimension $d^{\prime }$ is equal to (or larger than) the dimension d, the representation h can achieve perfect reconstruction by copying the input vector without discovering any useful information. To neglect the constraints for the dimension $d^{\prime }$, the denoising autoencoder (DAE) yields a more stable and robust result. 

The underlying idea of the denoising autoencoder is that vector h is yielded from the corrupted input $\tilde{x}$, which is constructed from input x by a stochastic mapping, $\tilde{x}∼q_{D}(\tilde{x}|x)$. Then, the noising input $\tilde{x}$ is mapped by the autoencoder $h=f_{\theta }(\tilde{x})$ and reconstructed $z=g_{\theta^{\prime }}(h)$. The reconstruction error is maintained as the distance between x and z, as above, when training the denoising autoencoder. Figure 4c shows the procedure of the DAE network. The network is forced to learn knowledge from the noise input to correct and reconstruct the clear input. Hence, the extracted feature from input x is output vector h from the hidden layer of the DAE network. This is because the input vector can be reconstructed from h, which means the vector h contains rich information about the raw input data. Moreover, the mathematical analysis shows that the reconstruction error criterion of the autoencoder satisfies the infomax principal of Linsker [24]. For extracting deeper or higher order information, the stacked denoising autoencoder is integrated with a greedy layer-wise strategy, i.e., a generative model with several layers of hidden causal variables. The stacked denoising autoencoder is formed by removing the output layer of the trained autoencoder and feeding the output h of the hidden layer to the next autoencoder. The output is thereby mapped from the previous layer with the aspiration of a better representation. 

With the iterative execution of this process by several DAE layers, a deep neural network with a desirable structure can be obtained. Consequently, each hidden layer produces a new representation that is more abstract than the previous layer. After that point, the fine-tuning of the whole network is performed using gradient descent in a supervised manner to obtain the ultimate criterion of interest for classification.

### 3.2. Fine-Tuning Phase for Classification

In the last phase of the training process, the proposed NMEC-DNN is trained end to end using supervised learning to simultaneously fine-tune the network parameters. The parameters except the final layer of the network are initialized from the pre-trained autoencoder as discussed above. The parameters are then simultaneously fine-tuned to minimize the error between the predicted value and the target label using the gradient descent method. In this study, the faults belong to eight classes: BND, BCO, BCI, BCR, BCOI, BCOR, BCIR, and BCOIR. Each class is independent and not mutually exclusive; e.g., one acoustic emission signal can concurrently contain both an outer and inner fault, which will be classified as a defect in the outer-inner raceways (BCOI). 

To achieve this objective, the target label of each class must exhibit the intrinsic relationship between classes. Table 1 shows the output labels of the DNN. For this formulation, a group of three bits encodes the labels of eight classes of the bearing faults. The three bits are denoted as three labels: Outer (O), Inner (I), and Roller (R). For each fault type, values of the labels flip to a particular combination as given in Table 1. Accordingly, the three bits with values of zero represent the label for defect-free bearing, and the three bits having values of one represent the label for the defect on outer-inner-roller (BCOIR). Hence, the output layer has three nodes with respect to the three-bit output.

In this study, the NMEC is applied to the last layer of the deep neural network. Each signal input is associated with a subset of labels, which are nominal relevant labels; all remaining labels are irrelevant. After the last hidden layer of the DNN, the scores with respect to each label are produced. A label predictor typically converts label scores to binary using a thresholding technique [25]; i.e., once training of the neural network is finished, the output may be interpreted as a probability distribution. Then, the labels are sorted by the probabilities in decreasing order, and a threshold is chosen to produce the best classification performances for each pair of successive positive labels. The threshold is associated with rank-loss [26] for backpropagation. However, it has been asserted that none of the convex loss functions or the discontinuities are consistent with respect to the rank-loss function, which is not suitable for a problem of non-mutually exclusive classes.

Consider an unknown instance x of a combined fault signal BCOI, which can be assigned in both classes BCO and BCI, where BCO and BCI are two non-mutually exclusive (dependent) classes. The mutual information between the two classes, BCO and BCI, is denoted as $I(BCO_{x}:BCI_{x})$. From the definition of Kullback–Leibler divergence, this mutual information is the relative entropy between the joint and the marginal product distributions. Consider that the random variable $\left(\hat{BCO},\hat{BCI}\right)$ is independent, but it has the same marginal as (BCO,BCI); i.e., $P(\hat{BCO},\hat{PCI})=P(BCO)P(BCI)$. Then,

(4) $$I(BCO:BCI)=D(BCO,BCI∥\hat{BCO},\hat{BCI})$$

where $D(A∥B)≜\sum_{x}\mathrm{Pr}[A_{x}]\log\frac{\mathrm{Pr}[A_{x}]}{\mathrm{Pr}[B_{x}]}$ is the Kullback–Leibler divergence. $\mathrm{Pr}[A_{x}]$ is the probability of a proposition {A=x}, where A is a random variable. From (4), the relation between the Kullback–Leibler divergence and cross-entropy yields

(5) $$I(BCO:BCI)=H((BCO,BCI);(\hat{BCO},\hat{BCI}))-S(BCO,BCI)$$

where $S(BCO,BCI)≜-\sum_{x}\mathrm{Pr}[BCO_{x},BCI_{x}]\log\mathrm{Pr}[BCO_{x},BCI_{x}]$ is the joint entropy of the joint distribution $\mathrm{Pr}[BCO_{x},BCI_{x}]$. The first element of the right-hand part of (5) is given by

(6) $$H(A;B)≜-\sum_{x}\mathrm{Pr}[A_{x}]\log\mathrm{Pr}[B_{x}]$$

which is defined as the cross-entropy between two distributions, $\mathrm{Pr}[A_{x}]$ and $\mathrm{Pr}[B_{x}]$. In formal information theory terms, this measures the average number of bits needed to identify events that occur with probability $\mathrm{Pr}[A_{x}]$, specifically if a coding scheme is used that is optimal for the probability distribution,$\mathrm{Pr}[B_{x}]$. Here, $\mathrm{Pr}[A_{x}]=\mathrm{Pr}[BCO_{x},BCI_{x}]$ and $\mathrm{Pr}[B_{x}]=\mathrm{Pr}[{\hat{BCO}}_{x},{\hat{BCI}}_{x}]$. Then, from (6), the cross-entropy of the joint distribution of $\mathrm{Pr}[BCO_{x},BCI_{x}]$ and the respected $\mathrm{Pr}[{\hat{BCO}}_{x},{\hat{BCI}}_{x}]$ are given as (7).

(7) $$H((BCO,BCI);(\hat{BCO},\hat{BCI}))=-\sum_{x}\mathrm{Pr}[BCO_{x},BCI_{x}]\log\mathrm{Pr}[{\hat{BCO}}_{x},{\hat{BCI}}_{x}]$$

This cross-entropy presents the uncertainty of whether instance x belongs to both BCO and BCI classes. $\mathrm{Pr}[{\hat{BCO}}_{x},{\hat{BCI}}_{x}]$ is obtained from the output of the front layer next to the last layer. A similar analysis is applied for each pair of joint distributions, as shown in Figure 4d. Then, the loss function is defined based on the cross-entropy function as:

(8) $$L(y,\hat{y})=-{\sum_{j}y}^{\left(j\right)}\log{\hat{y}}^{\left(j\right)}$$

The log-loss measures the uncertainty of the probabilities of classification model $\hat{y}$ by comparing them to the ground truth label, y. A perfect classifier would have a log-loss of precisely zero; a less ideal classifier would have progressively larger log-loss values. 

Here, we focus only on the probability of a data point belonging to a class. The real values from the network are first fed into the sigmoid function σ(⋅) that squashes the real-valued output to [0,1]. The model prediction becomes $\hat{y}(h)=\sigma (W^{T}h_{L}+b)$, where $\hat{y}$ is the predicted value from the output layer, and $h_{L}$ is the output of the last hidden layer of NMEC-DNN. For instance, the predicted value from the output layer or the model probability of assigning label m is denoted as ${\hat{y}}^{\left(m\right)}$. Here, $y^{\left(m\right)}\in \{0,1\}$ is the target value or a binary indicator of whether label m is the correct classification for an instance. Additionally, M is the number of possible labels, and the cross-entropy loss is defined based on the sigmoid function as:

(9) $$L_{\theta }(y,\hat{y})=\frac{1}{M}\sum_{m=1}^{M}\left[-y^{\left(m\right)}\log{\hat{y}}_{\theta }^{\left(m\right)}-(1-y^{\left(m\right)})\log(1-{\hat{y}}_{\theta }^{\left(m\right)})\right]$$

It is straightforward to show $\nabla L(W)=\frac{1}{M}\sum_{m}h.({\hat{y}}_{\theta }^{\left(m\right)}-y)$ and $\nabla L(b)=\frac{1}{M}\sum_{m}\left({\hat{y}}_{\theta }^{\left(m\right)}-y\right)$ for the output layer. This gradient is the only requirement for optimization. Moreover, the use of the cross-entropy loss avoids learning slowdown in many neural networks because the derivation of the loss function only depends on the output error instead of the derivation of the activation function.

## 4. Experimental Results and Discussion

### 4.1. Data Acquisition

**Figure 6:**
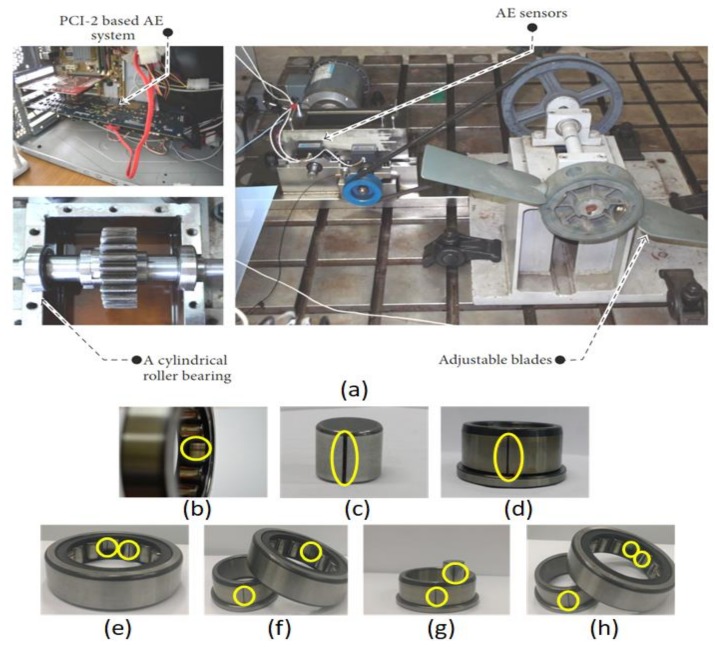
(**a**) Data acquisition system setup and the types of bearing defects: (**b**) BCO (outer raceway); (**c**) BCR (roller element); (**d**) BCI (outer raceway); (**e**) BCOR (outer–roller defect); (**f**) BCOI (outer–inner defect); (**g**) BCIR (inner–roller defect); (**h**) BCOIR (outer–inner roller defect) [27,28].

The algorithm was evaluated by applying it to the bearing fault data collected using a self-designed test rig, as shown in Figure 6. The cylindrical roller-element bearings (type FAG NJ206-3-TVP2) were installed in both shafts of the gearbox. The counter shaft was coupled with a three-phase motor for driving. A gearbox with a ratio of 1.52:1 was used to transmit the power from the counter shaft to the main shaft. The main shaft is connected with a bell and pulley to rotate the propeller, which can be used to vary load on the bearing. The bearing rotating speed was measured using a displacement transducer attached to the main shaft. An acoustic emission (AE) sensor (type PACWSα) was placed on the bearing housing to record the AE signals that indicated the state of the bearing health condition.

A total of eight types of bearing signals were recorded, including signals for seven types of bearing faults (BCO, BCI, BCR, BCOI, BCOR, BCIR, and BCOIR) and a healthy bearing, i.e., bearing with no defect (BND). For each type of fault, the signal was recorded at a rotational speed of 500 r/min. The duration of each signal is 1-s and was recorded at a sampling rate of 250 kHz using a PCI-2 system. According to the parameters of the bearing and the rotational speed of the shaft, the values of the defect frequencies of BPFO, BPFI, and BSF are 43.7 Hz, 64.6 Hz, and 20.7 Hz, respectively.

### 4.2. Experimental Results

Each 1-sec signal is a vector of size 250,000 corresponding to the sampling rate of 250 kHz. The AE signal cannot be used directly as input by the NMEC-DNN, as it would make training practically unfeasible. Therefore, we use feature extraction and consider two different scenarios. The AE signals are divided into 500 and 1000 segments each. When a 1-sec AE signal is segmented into 500 and 1000 segments, then the length of each segment is 500 and 250 samples, respectively. For each segment, different features are calculated, which are then grouped into a feature vector that is used as input to the neural network. The feature vectors are homogeneous, i.e., each feature vector consists of values of a single feature for all the segments. For example, a feature vector might contain 500 RMS values, one for each of the 500 segments of the AE signal. These features include the root mean square (RMS), skewness, crest factor, kurtosis, clearance factor, and entropy [29]. Thus, the NMEC-DNN is provided with inputs of two different sizes, i.e., 500 and 1000. 

The dataset for evaluating NMEC-DNN consists of 600 AE signals of 1-sec duration each, for each bearing with a single fault (BCO, BCI, BCR, and BND), i.e., a total of 2400 AE signals. These 2400 AE signals are divided into three subsets containing 2000 AE signals for training, 200 AE signals for validation, and 200 AE signals for testing. The data for testing the combined mode faults consists of 200 AE signals (50 AE signals for each type, BCOI, BCOR, BCIR, and BCOIR). As mentioned earlier, each of these AE signals is divided into (1) 500 segments, and (2) 1000 segments. Feature vectors are then constructed for all the AE signals by calculating the aforementioned features for each segment of the AE signal, and these feature vectors are used as inputs to the NMEC-DNN.

The parameters of NMEC-DNN are determined using state-of-the-art methods to maximize the accuracy. For the case of 500 inputs, the DNN is set up with three hidden layers, and the number of nodes in each layer is 600, 300, and 50. The SDAE activation function is the sigmoid function, and the deactivation phase uses the affine function. The stacked denoising autoencoder with the stochastic gradient descent algorithm is used to minimize the reconstruction error. For the case of 1000 inputs, the network is set to five hidden layers and the number of nodes in each layer is 1400, 600, 400, 100, and 50, respectively. 

**Figure 7:**
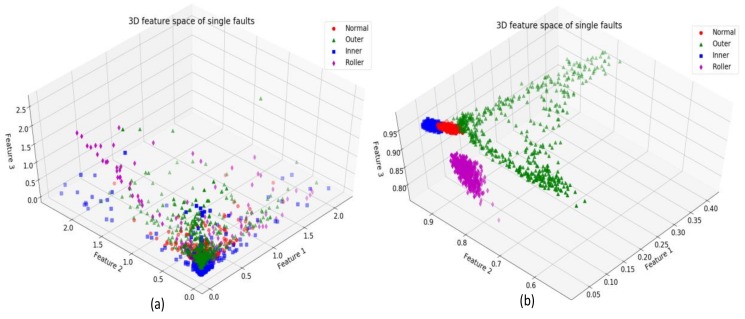
Feature space of the single faults: (**a**) before using stacked denoising autoencoders (SDAE) training and (**b**) after using SDAE training.

Figure 7 depicts the three-dimensional (3D) feature spaces of some features for the cases before and after using SDAE as the method for extracting and compressing information. The features extracted by SDAE show better separation and clustering for different fault types. Samples belonging to the same class are more closely clustered, whereas samples belonging to different classes can be easily discriminated as shown in Figure 7b. Whereas, before SDAE training, the features of different classes overlap each other making it difficult to distinguish those fault classes using these features as shown in Figure 7a. 

Among the proposed features that are used as inputs to NMEC-DNN, the RMS, which represents the average power of the AE signal and is effective in discovering incipient defects. The RMS value increases as the AE activity increases in a bearing with increasing deterioration. When each feature is considered separately, the RMS provides the most significant contribution to the classification performance. In contrast, the features, such as the kurtosis, crest factor, skewness, clearance factor, and entropy, show a lower contribution to the detection performance. To improve the classification accuracy, a more efficient approach is needed to incorporate the multi-featured information for a more consistent diagnostic performance. Thus, RMS is used as the primary feature because it provides the most useful information about the bearing faults. The other features are selected for use along with the RMS to provide additional defect information for improving the classification performance. 

The results illustrate that the kurtosis provides the best result owing to its ability in capturing the spikiness of the fault signal, which increases with increasing AE activity. The kurtosis measures the sharpness of the peaks and is helpful in identifying transient and spontaneous events within the AE signal. Since the kurtosis has a high sensitivity to incipient defects it is not very stable. Therefore, it can be combined with another feature to enhance its ability to characterize a signal. Hence, when the kurtosis and RMS are applied together, the performance is increased significantly to a maximum of 100% for the single mode defects and to 95% for the combined mode defects. The results of classification by NMEC-DNN are given in Table 2.

The results of the proposed method are compared with a set of SVMs that are trained in the OAA multi-class framework using the same settings as the proposed method. The SVMs show relatively poor diagnostic performance in the case of combined faults with a maximum accuracy of 49%, although they work well for the single fault with an accuracy of 92%. 

**Figure 8:**
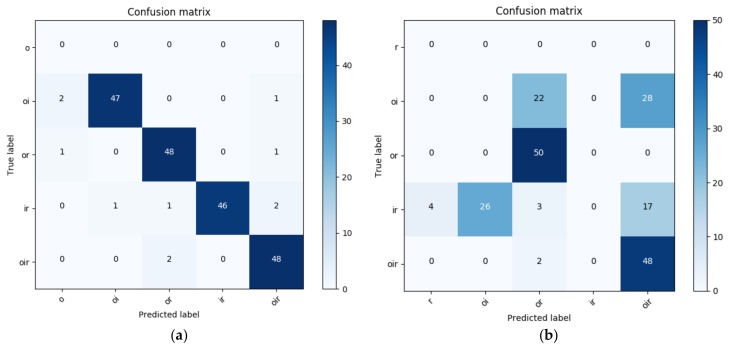
Confusion matrixes for (**a**) NMEC-DNN classification; (**b**) SVM classification with root mean square (RMS) and kurtosis features.

Meanwhile, NMEC-DNN shows better performance in diagnosing both single and combined faults with a maximum classification accuracy of 100% and 95%, respectively. The confusion matrices, which show the results of NMEC-DNN and SVM classification for each class using the RMS and kurtosis features, are shown in Figure 8. The NMEC-DNN shows better performance in the classification of most of the combined fault classes. However, the SVM mostly misclassifies the two classes, BCOI and BCOIR. In this case, when the bearing has the combined defect in all components (outer, inner, roller; Figure 2g), the first harmonics of 2×BSF and BPFO are very close to each other. Moreover, 2×BSF has a small amplitude and is easily covered by the side-band of BPFO. The higher harmonics of these defect frequencies, which contain minimal energy, are commonly overwhelmed by the noise and higher-level vibration of the macro-structure components. Thus, the two signals cannot be clearly differentiated, as shown in Figure 2d,g.

Moreover, in the combined fault cases the tightness-of-fit of the components also affects the actual frequency and there are differences between the measured frequencies on account of the random slip. The other case of misclassification is between BCIR and BCOI, which is due to the interaction between the fault signals of the combined fault. 

In practice, when the multiple combined modes appear in the bearing, the different types of fault signals interact with each other. The transformation of the problem of multi-class classification into three two-class classification sub-problems defines the model in which each class is independently exclusive and cannot interact with other classes. The disadvantage of this transformed model illustrates that the SVM method may not have been the most accurate methodology for all situations. It does not naturally handle non-mutually exclusive classes or generate a proper probability estimation. In contrast, NMEC-DNN used with the probability model can capture and model the dependencies between combined fault classes better than the multi-class SVM.

## 5. Conclusions

A reliable methodology for combined bearing fault diagnosis was proposed in this paper. This methodology builds a deep neural network based on the stacked denoising autoencoder aggregate non-mutually exclusive classifier. Owing to the layer’s SDAE architecture, the proposed NMEC-DNN demonstrated its ability to automatically learn features from single mode faults and to effectively classify the combined mode faults even though the combined mode data were not used during the training stage of the network. This strategy helps in building a more practical classification methodology with lesser data requirements and better diagnostics performance under different operating conditions. Moreover, it provides an advanced failure warning in the rotary machine operation and reduces unexpected failures in real applications. The experimental results using a bearing dataset indicated that the proposed methodology achieves a maximum classification accuracy of 95%. The results also illustrated that NMEC-DNN yields significantly better performance compared to the set of SVMs for multi-label classification. In future work, our research will focus on modifying the NMEC-DNN structure for suitability to large-scale input data.

## Figures and Tables

**Table 1:** Encoding the output label of DNN.

Types of Bearing Faults	Labels		
	O	I	R
Normal	0	0	0
Outer	1	0	0
Inner	0	1	0
Roller	0	0	1
Outer + Inner	1	1	0
Outer + Roller	1	0	1
Inner + Roller	0	1	1
Outer + Inner + Roller	1	1	1

**Table 2:** Results of Classification.

		500 Inputs		1000 Inputs	
		Single	Combined	Single	Combined
Root Mean Square (**RMS**)	**SVMs**	–	–	–	–
	**NMEC-DNN**	**88–91%**	**87–89%**	**93–95%**	**88–90%**
**Kurtosis**	**SVMs**	–	–	–	–
	**NMEC-DNN**	81–82%	78–80%	84–85%	81–83%
**Crest factor**	**SVMs**	–	–	–	–
	**NMEC-DNN**	65–68%	63–64%	73–74%	69–71%
**Clearance factor**	**SVMs**	–	–	–	–
	**NMEC-DNN**	62–65%	58–61%	66–68%	61–63%
**Skewness**	**SVMs**	–	–	–	–
	**NMEC-DNN**	72–76%	71–74%	74–76%	73–75%
**Entropy**	**SVMs**	–	–	–	–
	**NMEC-DNN**	77–79%	69–72%	78–80%	76–78%
**RMS + Crest factor**	**SVMs**	90%	44.5%	83%	50%
	**NMEC-DNN**	84–86%	80–83%	87–89%	83–84%
**RMS + Skewness**	**SVMs**	84.5%	42.5%	69%	26%
	**NMEC-DNN**	60–63%	50–54%	76%	74%
**RMS + Clearance factor**	**SVMs**	98%	46.5%	87.5%	33%
	**NMEC-DNN**	72–75%	69–72%	80–84%	70–74%
**RMS + Entropy**	**SVMs**	83.5%	20.5%	82.5%	26%
	**NMEC-DNN**	65–68%	60–63%	74–77%	70–73%
**RMS + Kurtosis**	**SVMs**	92%	46%	92.5%	49%
	**NMEC-DNN**	**91–93%**	**88–91%**	**99–100%**	**93–95%**
